# The Ready Reference Handbook of Skin Diseases

**Published:** 1901-12

**Authors:** 


### BOOK REVIEWS.

THE READY REFERENCE HANDBOOK OF SKIN DISEASES. By GEORGE THOMAS JACKSON, M. D., Chief of Clinic and Instructor in Dermatology, College of Physicians and Surgeons, New York. New (4th) edition, thoroughly revised. In one 12mo volume of 617 pages, with 82 engravings and 3 colored plates. Philadelphia and New York: Lea Brothers & Co. 1901. [Price, cloth, $2.75 net]

The prompt appearance of the fourth edition of this work, first published in 1892, is very strong testimony to its excellence and value. Dr. Jackson makes no attempt to conform to conventional classifications, but continues to arrange his subjects alphabetically, which is certainly more convenient for quick reference and calculated to avoid confusion in the mind of the enquirer. The author's power to describe a disease in a few short, clear sentences is probably unrivaled. We know of no easier inroad into the general subject of dermatology than is furnished by this admirable book. The author most sensibly avoids the prolix discussion of mere theories, and says in forcible words all that is really necessary to give the reader a firm grasp upon the subject treated.

New sections have been added to the work which are important, since they make the general subject clearer, although, in order to save space, the author has omitted a considerable portion of the old text. This may be an improvement in some respects; it is otherwise so far as the pronunciation is concerned, which was a great help to students. Among the new illustrations we most especially commend the one furnished by Dr. Hubbard relating to smallpox and representing the stages of maturation and umbilication present in lesions so large, the condition usually asserting itself during the stage of vesiculation.

The work, as a whole, as the present writer takes occasion to say for the fourth time, is one of the best and most useful of its class. We hope to see another edition hereafter, containing all the old and new matter in combination. Beyond this, nothing could be asked. G. W. W.

